# A Case of Stricture in the Male Urethra of Twenty-Five Years’ Standing

**Published:** 1877-07

**Authors:** R. W. Westmoreland

**Affiliations:** Atlanta, Ga.


ATLANTA

MEDICAL AND SURGICAL JOURNAL

VOL. XV.] JULY—1877. [No. 4

### Original Communications.

### A CASE OF STRICTURE IN THE MALE URETHRA OF TWENTY-FIVE YEARS STANDING—EXTERNAL URETHROTOMY—CURE.

BY R. W. WESTMORELAND, M.D., ATLANTA, GA.

In reporting the following case, I do not propose to present anything new, but certain peculiar features that may prove of interest to some. As is illustrated in this patient, simple affections, comparatively, are allowed, through neglect or ignorance, to develop most serious conditions, requiring heroic measures for relief.

Mr. W., of Campbell county, aged 58, the subject of the following report, from mechanical causes became the victim of stricture. According to his statement, when micturition first became difficult, he was advised by a physician to contract some venereal disease, in order that the cure of the original affection might be facilitated! In the advised treatment his case was of course made worse by additional cause of irritation, and which was still further increased by a false passage and the frequent attempts at introducing catheters. The stricture was complete so far as the introduction of instruments into the bladder was concerned.

In this state of affairs, his condition was deplorable. At each occasion of urination he would be subjected to the most distressing pain. His whole frame would tremble with the effort, and he would finally accomplish the act—was left as helpless as a child.

Finally he came under the treatment of a more rational physician, who attempted assiduously the introduction of filliform bougies, in the hope of relieving by gradual dilatation. His failure in this was owing, no doubt, to the tortuousness of the canal, resulting from the false passage before spoken of. At least this assisted in the difficulty, while there were other troublesome factors, to be spoken of. Having undergone this manner of treatment for six weeks or two months without any benefit, he lost hope. Gradually he failed in strength, and severe rigors, indicating the fatal progress of his affliction on the constitutional powers, supervened. He was an exhibition of an almost complete wreck of a once strong and vigorous man. On account of the implication of the kidneys in the irritation, he had to constantly dose himself with spirit nitre and buchu; otherwise his difficulty in micturition would be increased by the acrid and loaded condition of the urine, resulting from abnormal renal action.

In this condition, after suffering more or less for twenty-five years, he came under my treatment. Upon careful exploration, I found that it was impossible to introduce any kind of an instrument through the obstruction into the bladder; and both from my own experiment and the efforts that had been made in this line before me, I decided to abandon all idea of dilatation, and resort to incision.

I discovered a circumscribed tumor at the spongy portion of the urethra, just anterior to the subpubic curve. A perceptible increase of size in the tumor could be noticed on micturition. No fluctuation was perceptible, but upon the forcible injection of oil during my effort to introduce a bougie, much relief was afforded in urinating, and the volume of the tumor was much diminished.

A grooved director being passed down to the anterior border of the tumor, as far as it could be insinuated, and the scrotum pulled well up on the pubis, the patient being in the lithotomy position, a free incision was made down on the point of the instrument. The tissue of the tumor was found to be very dense and gristley, and contained no urine, as far as I could discover. The stricture, situated just a little in front of the anterior margin of this tumor, was about half an inch in extent, dense, cartalaginous in structure, and very tough. The director was found to be not in the regular channel, but passed out into a false passage just anterior to the point of constriction. In the tumor was found a calculus about the size and shape of a small almond kernel, resting in a kind of cul-de-sac of the dilated urethra. This was removed, and, with a meatome, the the stricture was opened from behind forwards, and the director reintroduced through the natural channel into the bladder without further obstruction.

The peculiar feature that presented itself to my mind at this stage of the operation was the direction of the director into this false passage. When the stricture had been incised, the point of the instrument could pass as readily through the regular channel as it before had passed through the false one to the point where the stone was situated; but it could by no manner of persuasion be passed by the stone until it had been removed. Furthermore, no difficulty afterward occurred in reaching the bladder, the instrument seeming to have always struck the regular passage, and to have glided clear of the false one altogether. Had this not been the case, the opening at the point where the stone was situated would not so accurately have received the beak of the sound, and difficulty, for awhile, would have been experienced in its introduction.

It is needless to say that instant relief was the result. A catheter of suitable size was easily introduced, and, for the first time in ten years, his urine was drawn off. Up to the present time he has done splendidly; the incision healing kindly, and no unfavorable symptoms having made their appearance.

The gratitude of the old gentleman could not be expressed in words—he could only *look* it. The blank despair that had settled like an incubus upon his mental being had given place to ecstatic joy and hope. That which might have been done years before, was done at last, though after untold suffering and the most demoralizing misery.

It is such instances as this that should impress upon us the inutility, the injury of temporizing with any one mode of operating in stricture, or allowing foolish timidity to rob us of the golden fruits arising from a timely and rational surgical interference.

